# Renalase and Total Antioxidant Status in Relation to CCTA-Assessed Coronary Artery Disease Severity in Suspected Obstructive Sleep Apnea

**DOI:** 10.3390/antiox15070797

**Published:** 2026-06-26

**Authors:** Paweł Gać, Michał Fułek, Monika Michałek, Piotr Macek, Małgorzata Poręba, Helena Martynowicz, Rafał Poręba

**Affiliations:** 1 Department of Environmental Health, Occupational Medicine and Epidemiology, Wroclaw Medical University, Mikulicza-Radeckiego 7, 50-345 Wroclaw, Poland; 2 Centre of Diagnostic Imaging, 4th Military Hospital, Weigla 5, 50-981 Wroclaw, Poland; 3 Department and Clinic of Diabetology, Hypertension and Internal Diseases, Institute of Internal Diseases, Wroclaw Medical University, 50-556 Wroclaw, Poland; michal.fulek@umw.edu.pl (M.F.);; 4 Department of Diabetology and Internal Medicine, A. Falkiewicz Specialist Hospital in Wroclaw, Warszawska 2, 52-114 Wroclaw, Poland; 5 Department of Cardiology, Marciniak Lower Silesian Specialist Hospital, Fieldorfa 2, 54-049 Wroclaw, Poland; 6 Department of Biological Principles of Physical Activity, Wroclaw University of Health and Sport Sciences, 51-612 Wroclaw, Poland

**Keywords:** coronary artery disease, coronary computed tomography angiography, obstructive sleep apnea, renalase, total antioxidant status

## Abstract

Aim: The aim of this observational cross-sectional study was to evaluate whether blood renalase concentration, total antioxidant status (TAS), main cardiovascular risk factors, and obstructive sleep apnea severity are associated with the anatomical severity of coronary artery disease assessed by CCTA in patients with suspected OSA. Materials and methods: The study included 93 patients with suspected OSA. All patients were assessed for main risk factors for cardiovascular disease. Polysomnography was performed to verify the suspicion of OSA, as well as coronary computed tomography angiography (CCTA) with a systematic assessment of the severity of coronary artery disease using the CAD-RADS classification. Blood renalase concentration and total antioxidant status (TAS) were determined. Results: The apnea–hypopnea index (AHI) in the study group was 16.57 ± 17.17 /h. OSA was diagnosed in 73.1% of the study group. In CCTA examinations, significant coronary artery disease (CAD-RADS ≥ 3) was suspected in 22.6% of the subjects, including 16.1% classified as CAD-RADS 3, 4.3% as CAD-RADS 4, and 2.1% as CAD-RADS 5. Patients with AHI ≥ median were significantly more often classified as CAD-RADS ≥ 3 than patients with AHI < median. Patients with blood renalase concentration ≥ median were significantly less often classified as CAD-RADS ≥ 3 than patients with blood renalase concentration < median. Similarly, patients with TAS ≥ median were significantly less often classified as CAD-RADS ≥ 3 than those with TAS < median. Older age, higher systolic blood pressure, higher blood cholesterol levels, and lower TAS were independently associated with CAD-RADS ≥ 3 in logistic regression analysis. In multivariable regression analysis, higher pack-years of smoking, higher AHI, and lower blood renalase concentration were independently associated with lower TAS. Conclusions: Higher pack-years of smoking, higher AHI values, and lower blood renalase concentration were associated with lower total antioxidant status, which, along with older age, higher systolic blood pressure, and higher total cholesterol concentration, was independently associated with suspected anatomically significant coronary artery disease on CCTA.

## 1. Introduction

Ischemic heart disease, most commonly caused by atherosclerotic coronary artery disease (CAD), remains one of the principal causes of death, disability, and health-care burden worldwide. In the Global Burden of Disease cardiovascular analysis, ischemic heart disease had the highest global age-standardized disability-adjusted life-year rate among cardiovascular causes, underscoring its persistent epidemiological dominance despite improvements in cardiovascular prevention and treatment [1]. This burden is also highly relevant in Europe, where cardiovascular disease remains the leading cause of mortality and disability, with marked geographic and socioeconomic disparities across countries [2,3]. Because coronary atherosclerosis may remain clinically silent until an acute coronary event or may present with nonspecific symptoms, accurate identification of patients with suspected CAD remains a central objective of contemporary cardiovascular risk assessment and diagnostic pathways.

Coronary computed tomography angiography (CCTA) has become a central non-invasive anatomic imaging modality in the diagnostic evaluation of patients with suspected CAD. Contemporary guidelines recommend non-invasive anatomic or functional imaging as first-line diagnostic testing in appropriately selected patients with suspected chronic coronary syndromes, with CCTA particularly useful for excluding obstructive CAD and detecting non-obstructive coronary atherosclerosis [4,5]. Beyond the binary detection of stenosis, CCTA enables direct visualization of the coronary lumen, plaque burden, and the anatomic extent of disease, thereby supporting individualized risk stratification and preventive management. Its clinical relevance is further strengthened by randomized evidence showing that CT-based diagnostic strategies are a safe and clinically informative alternative to invasive coronary angiography in selected patients with stable chest pain [6]. Standardized reporting systems, particularly CAD-RADS, provide a structured framework for classifying the severity of CAD on CCTA and facilitate consistent communication of findings relevant to subsequent clinical decision-making [7].

Sleep health is increasingly recognized as an integral component of cardiovascular health rather than only a determinant of quality of life. This concept was formalized in the American Heart Association Life’s Essential 8 framework, in which sleep health was added to diet, physical activity, nicotine exposure, body weight, blood lipids, blood glucose, and blood pressure as a core metric of cardiovascular health [8]. Contemporary statements emphasize that sleep health is multidimensional and includes sleep duration, continuity, timing, regularity, daytime functioning, sleep architecture, and the absence of sleep disorders [9]. Among clinically relevant sleep disorders, obstructive sleep apnea (OSA) is particularly important in cardiovascular medicine because it is highly prevalent, frequently underdiagnosed, and characterized by recurrent upper-airway obstruction leading to intermittent hypoxemia, autonomic fluctuation, and sleep fragmentation [10,11]. These pathophysiological features have been linked to hypertension, metabolic dysregulation, arrhythmias, heart failure, stroke, and coronary artery disease, supporting the clinical relevance of evaluating CAD in patients with suspected OSA [10].

Redox imbalance has been proposed as one of the biological pathways linking sleep-disordered breathing with accelerated coronary atherosclerosis. Reactive oxygen and nitrogen species participate in physiological redox signaling, but excessive, sustained, or compartment-specific oxidative activity may promote endothelial dysfunction, reduced nitric oxide bioavailability, lipid oxidation, vascular inflammation, and plaque progression [12,13]. In CAD, these processes involve multiple enzymatic and cellular sources of reactive oxygen species, including NADPH oxidases, mitochondrial dysfunction, xanthine oxidase, and uncoupled endothelial nitric oxide synthase, with evidence from human coronary arteries supporting increased vascular oxidative activity in established disease [14,15]. In OSA, recurrent cycles of hypoxia and reoxygenation, sleep fragmentation, and sympathetic activation may further enhance oxidative stress and redox-sensitive inflammatory signaling, creating a biologically plausible interface between OSA and coronary vascular injury [16,17]. More broadly, sleep-related disorders and motor phenomena have also been linked to systemic inflammatory and oxidative stress profiles, as suggested by a systematic review on sleep bruxism and chronic inflammation and by polysomnography-based evidence connecting sleep bruxism with oxidative stress [18,19]. However, available clinical data remain heterogeneous, and oxidative stress markers do not consistently distinguish OSA patients with and without cardiovascular complications [20]. Therefore, global measures of antioxidant defense, such as total antioxidant status (TAS), may be useful for characterizing systemic redox balance in patients with suspected OSA and coexisting risk of CAD, while requiring cautious interpretation as integrative rather than disease-specific biomarkers.

Renalase is a circulating flavoprotein originally described as a kidney-derived enzyme involved in cardiovascular regulation, with additional expression in the heart, skeletal muscle, and other tissues [21]. Although its initially proposed catecholamine-metabolizing activity remains mechanistically debated, subsequent biochemical work has identified renalase as an α-NAD(P)H oxidase/anomerase, suggesting a potential link with intracellular redox homeostasis and nicotinamide nucleotide metabolism [22]. Experimental and clinical studies have also linked renalase with blood pressure regulation, endothelial dysfunction, ischemic cardiovascular disease, and myocardial injury, although the direction and biological meaning of circulating renalase changes may vary across populations and comorbid conditions [21,23,24]. In patients with suspected OSA, lower renalase concentrations have been associated with lower total antioxidant status (TAS), higher apnea–hypopnea index values, and higher blood pressure, supporting the hypothesis that circulating renalase may reflect, or partly participate in, systemic antioxidant defense [25]. However, the relationship between renalase, global antioxidant capacity, sleep-disordered breathing, and the anatomical severity of CAD assessed by CCTA remains insufficiently defined.

The objective of this observational cross-sectional study was to evaluate whether obstructive sleep apnea severity, circulating renalase concentration, total antioxidant status, and conventional cardiovascular risk factors are associated with the anatomical severity of coronary artery disease assessed by CCTA in patients with suspected OSA. We hypothesized that more severe sleep-disordered breathing and lower renalase concentration would be associated with reduced systemic antioxidant capacity, and that impaired antioxidant status would be related to more advanced CCTA-defined coronary artery disease. The novelty of the present study lies in the integrated assessment of polysomnography-derived OSA severity, circulating renalase, total antioxidant status, and standardized CCTA-based CAD severity using CAD-RADS in the same clinically relevant population of patients with suspected OSA.

## 2. Materials and Methods

This was an observational, cross-sectional study conducted in a single clinical cohort of adult patients with suspected obstructive sleep apnea and clinical indications for coronary computed tomography angiography. All clinical, polysomnographic, biochemical, and CCTA-derived variables were assessed at a single study time point, and no longitudinal follow-up or intervention was performed.

The study was conducted as part of a scientific project entitled “Sleep disorders assessed by polysomnography and non-invasive estimated risk of significant coronary heart disease in the population of patients with arterial hypertension” financed by the Wroclaw Medical University (project number in the Simple system STM.A100.20.141), which received a positive opinion of the Bioethics Committee at the Wroclaw Medical University (opinion no. KB 369/2020 of 17 June 2020).

The required minimum size of the study population was estimated using a sample size calculator. The following sample size estimation conditions were adopted: population size of 3,000,000 (the population size of the Lower Silesian macroregion in Poland), fraction size of 0.4 (averaged prevalence of cardiovascular disease in the Polish population), maximum error of 10%, and confidence level of 5%. The minimum required size of the study population was 92.

Inclusion criteria included age > 18 years, clinical suspicion of obstructive sleep apnea, clinical indications for coronary computed tomography angiography (CCTA), and willingness to participate in the study. Patients with previously diagnosed significant coronary artery disease, patients with a history of invasive cardiac or vascular surgery, a history of stroke, chronic renal failure, hyperthyroidism, hypothyroidism, and patients with insufficient CCTA quality were excluded. Exclusion criteria also included severe mental disorders that prevented polysomnography, taking medications that may affect the respiratory system, active cancer, and active inflammation. A total of 93 patients were included in the study. The general clinical characteristics of the study group are summarized in Table 1.

Patients who were consecutively included in the study underwent a questionnaire; basic anthropometric measurements (age, height, body mass, and body mass index (BMI)); blood pressure; laboratory tests (total cholesterol, triglycerides and glucose in blood; total antioxidant status (TAS) and renalase in blood); polysomnography; and coronary computed tomography angiography were performed. Based on the survey, anthropometric measurements, and standard laboratory tests, each patient was assessed for common risk factors for cardiovascular disease, such as obesity, hypertension, dyslipidemia, type 2 diabetes, and smoking.

Comorbidities and cardiovascular risk factors were defined according to standard clinical criteria. Overweight was defined as BMI ≥ 25.0 and <30.0 kg/m^2^, and obesity as BMI ≥ 30.0 kg/m^2^. Arterial hypertension was defined as a previous diagnosis of hypertension, current use of antihypertensive medication, or blood pressure values fulfilling the current diagnostic criteria during the study assessment. Dyslipidemia was defined as a previous diagnosis of dyslipidemia, current lipid-lowering therapy, or lipid values fulfilling standard diagnostic criteria. Type 2 diabetes mellitus was defined as a previous diagnosis of diabetes, current use of glucose-lowering therapy, or glucose values fulfilling current diagnostic criteria during the study assessment. Smoking status was assessed by questionnaire, and cumulative tobacco exposure was expressed as pack-years.

A polysomnographic (PSG) study was performed using NOXA1 recorders, without an adaptive night. PSG recordings were evaluated according to the American Academy of Sleep Medicine guidelines. Obstructive sleep apnea (OSA) was diagnosed based on the apnea–hypopnea index (AHI) on the polysomnographic study. The typical criterion for sleep apnea was an AHI of ≥5. The severity of OSA was also classified based on the AHI value. Patients with an AHI of 5–15 were diagnosed with mild OSA, those with an AHI of 15–30 were diagnosed with moderate OSA, and those with an AHI of 30 or higher were diagnosed with severe OSA.

Computed tomography (CT) was performed using a standard coronary computed tomography angiography (CCTA) protocol using a dual-source 384-slice SOMATOM Force CT scanner (Siemens Healthcare, Erlangen, Germany). The acquired images were evaluated by a certified cardiovascular radiologist with the EBCR Diploma (European Board of Cardiovascular Radiology Diploma), EACVI CCT exam (European Association of Cardiovascular Imaging—Cardiac Computed Tomography examination), and 15 years of clinical experience. The risk of significant coronary artery disease was assessed based on the coronary artery calcium score (CACS). Coronary artery disease severity was determined based on the Coronary Artery Disease Reporting and Data System (CAD-RADS), where 0-documented absence of coronary artery disease (CAD), 1-minimal non-obstructive coronary artery disease (maximum stenosis: 1–24%), 2-mild non-obstructive coronary artery disease (maximum stenosis: 25–49%), 3-moderate coronary artery disease (maximum stenosis: 50–69%), 4-severe coronary artery disease (maximum stenosis: 70–99%), and 5-complete coronary artery occlusion. Additionally, left ventricular systolic function parameters were assessed.

Total antioxidant status (TAS) was assessed using an enzyme-linked immunosorbent assay (ELISA) (no. 709001, Cayman Chemicals, Ann Arbor, MI, USA). Blood renalase levels were also determined using an ELISA kit (no. E3109Hu, Bioassay Technology Laboratory, Shanghai, China). ELISA tests were performed according to the manufacturers’ instructions.

Statistical analysis was performed using the “Dell Statistica 13” statistical program (Dell Inc., Tulsa, OK, USA). For quantitative variables, arithmetic means (X) and standard deviations (SD) of the determined parameters were calculated. Variable distribution was assessed using the Lilliefors and Shapiro–Wilk tests. For variables with non-normal distribution, non-parametric methods were used where appropriate. Given the substantial variability of circulating renalase concentrations, renalase was also analyzed using median-based subgrouping, logistic regression, and ROC analysis with sensitivity and specificity estimation. Results for ordinal and categorical (nominal) variables were expressed as percentages. For comparative analysis, the maximum likelihood chi-square test was used for further statistical analysis for independent categorical variables. Multivariate and logistic regression analyses were performed to determine the relationship between the studied variables. The parameters of the model obtained in the regression analysis were estimated using the least squares method. The sensitivity and specificity of the obstructive sleep apnea severity index AHI, total antioxidant status TAS, and blood renalase concentration as exploratory discriminators of CAD-RADS ≥ 3 on CCTA were assessed. Results at the level of *p* < 0.05 were considered statistically significant.

## 3. Results

In the study group, the diagnosis of obstructive sleep apnea was confirmed in 73.1% of patients. In 26.9% of patients, OSA was excluded based on polysomnography. Among patients with OSA, 33.3% were diagnosed with mild OSA, 19.4% with moderate OSA, and 20.4% with severe OSA. The average AHI in the study group was 16.57 ± 17.17/h. The mean AHI in patients with OSA was 21.99 ± 17.11/h, and was statistically significantly higher than in patients without OSA, in whom it was 1.82 + 1.48/h (*p* < 0.05). Among the main cardiovascular risk factors, the highest prevalence in the study group was observed for obesity (44.1%), arterial hypertension (36.6%) and dyslipidemia (30.1%). Data on obstructive sleep apnea and cardiovascular risk factors in the study group are presented in Table 1.

In CCTA, the mean coronary artery calcium score was 102.11 ± 200.92, with a median value of 3.80 and a range of 0 to 947.3. No risk of significant coronary artery disease based on CACS was found in 43.0% of patients. 17.2% of patients had a moderate risk of significant CAD, and 8.6% had a significant risk of significant CAD. Based on the angiographic phase of coronary CTA, coronary artery disease was excluded in 41.9%. Significant coronary artery disease was suspected in 22.6% (CAD-RADS ≥ 3). Coronary artery occlusions were visualized in 2.1% (CAD-RADS 5), coronary artery stenosis exceeding 70% (CAD-RADS 4) in 4.3%, and coronary artery stenosis of 50–70% (CAD-RADS 3) in another 16.1%. The study group had a normal left ventricular ejection fraction, averaging 69.16 ± 7.03%, with a range of 52–83%. The basic CCTA test results are summarized in Table 2.

The mean blood renalase concentration was 190.33 ± 212.59 ng/mL, and the mean TAS was 1.16 ± 0.33 mM. The median blood renalase concentration was 64.03 ng/mL. The determined blood renalase concentrations ranged from 2.39 ng/mL to 790.48 ng/mL, and the determined TAS ranged from 0.47 mM to 2.61 mM, Table 1.

Table 3 presents a comparative analysis of the severity of coronary artery disease assessed by multidetector computed tomography angiography in the study subgroups distinguished based on the criteria of OSA, blood renalase concentration and TAS.

Patients with AHI ≥ 10.10/h were more likely to have significant coronary stenoses on CCTA than patients with AHI < 10.10/h (CAD-RADS ≥ 3: 31.9 vs. 13.0%, *p* < 0.05). Similarly, in patients with blood renalase concentration < 64.03 ng/mL, the image of coronary arteries on CCTA significantly more often met the diagnostic criterion of CAD-RADS ≥ 3, compared to patients with blood renalase concentration ≥ 64.03 ng/mL (CAD-RADS ≥ 3: 32.6 vs. 12.8%, *p* < 0.05). Patients with TAS < 1.14 mM were significantly more often to be classified as CAD-RADS ≥ 3 than those with TAS ≥ 1.14 mM (CAD-RADS ≥ 3: 36.4 vs. 10.2%, *p* < 0.05).

A regression analysis was performed for two different dependent variables: first for the dichotomous variable CAD-RADS ≥ 3 (yes/no) using the logistic method, and then for the quantitative variable TAS using the backward stepwise multivariable method. The results of regression analysis in the study group are presented in Table 4. A final model obtained for CAD-RADS ≥ 3 as a dependent variable was: logit CAD-RADS ≥ 3 = −17.01 + 0.09 age + 0.06 systolic blood pressure + 0.03 blood total cholesterol concentration-4.64 TAS. It has been shown that older age, higher systolic blood pressure, higher blood total cholesterol concentration and lower TAS were independent predictors of CAD-RADS ≥ 3 in CCTA, Table 4.

**Figure 1:**
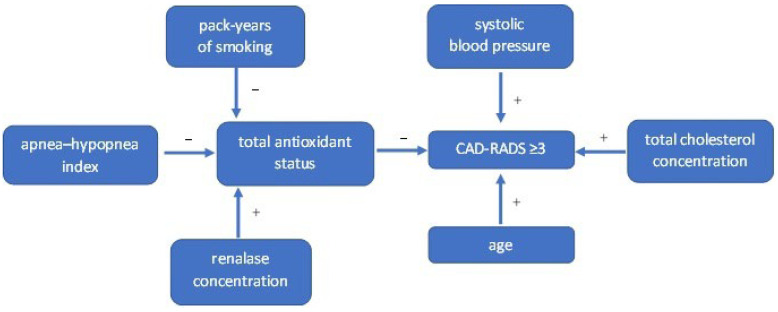
Diagram summarizing the regression analysis results.

Whereas, a final model obtained for TAS as a dependent variable was: TAS = 1.328 − 0.003 pack-years of smoking −0.005 AHI + 0.001 blood renalase concentration. In this multivariable regression analysis model, higher pack-years of smoking, higher AHI, and lower blood renalase concentration were independently associated with lower TAS, Table 4. A graphical summary of the regression analysis results is provided in Figure 1.

**Figure 2:**
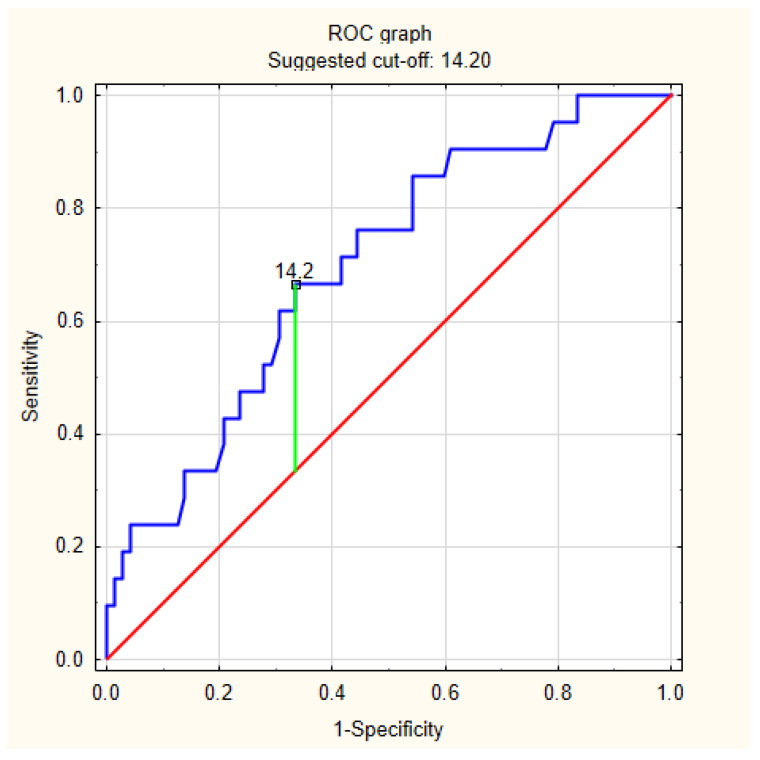
ROC curve of discrimination of CAD-RADS ≥ 3 based on AHI (/h) values in the study group.

**Figure 3:**
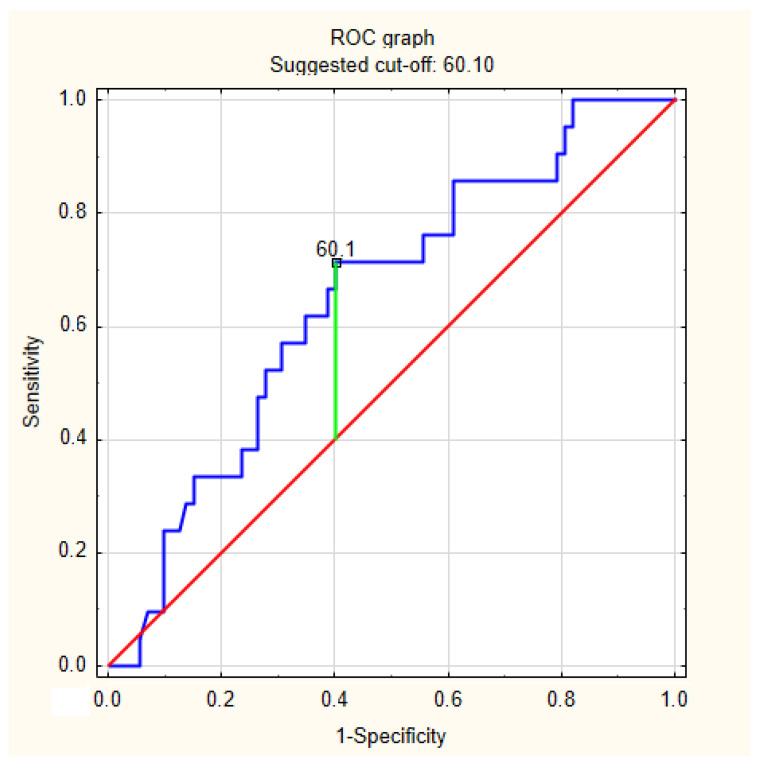
ROC curve of discrimination of CAD-RADS ≥ 3 based on renalase (ng/mL) values in the study group.

**Figure 4:**
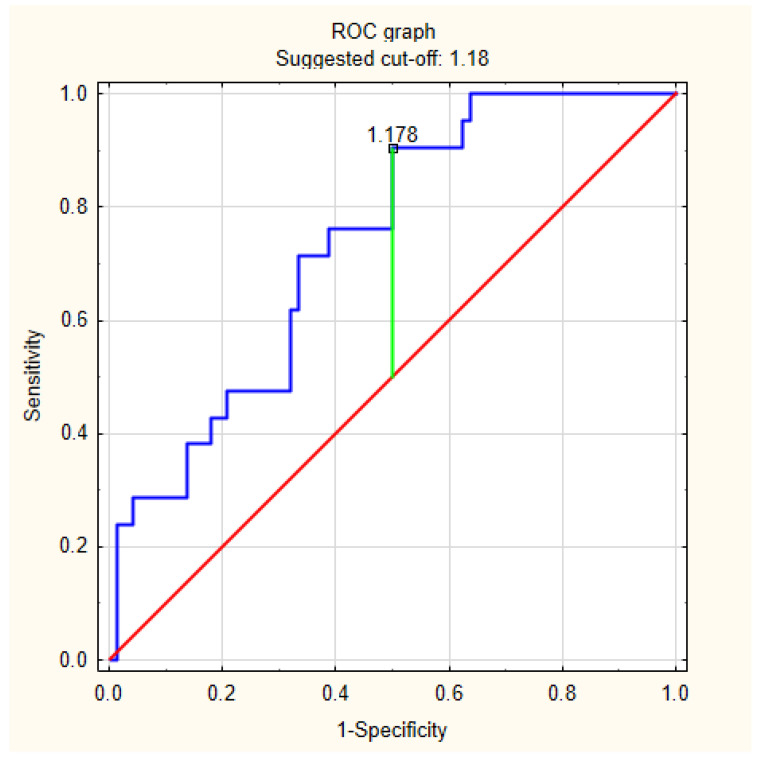
ROC curve of discrimination of CAD-RADS ≥ 3 based on TAS (mM) values in the study group.

The exploratory analysis of the discriminatory performance of selected variables for CAD-RADS ≥ 3 included AHI, blood renalase concentrations, and TAS. Using ROC curve analysis, ROC-derived thresholds with the best discriminatory performance in the present cohort were identified: AHI > 14.2/h, renalase concentration in blood < 60.10 ng/mL and TAS < 1.18 mM. The obtained ROC curves are shown in Figure 2, Figure 3 and Figure 4.

The sensitivity and specificity of discriminators obtained based on values commonly considered diagnostic (AHI > 5/h as a criterion for diagnosing OSA) and obtained based on ROC curve analyses are presented in Table 5.

The highest sensitivity of CAD-RADS ≥ 3 discrimination, which was 66.7%, was observed for the AHI > 14.2/h criterion. The highest specificity of CAD-RADS ≥ 3 discrimination, which was 90.5%, was observed for the AHI > 5/h criterion. Overall, the highest accuracy of CAD-RADS ≥ 3 discrimination was observed for the AHI > 14.2/h criterion and was 65.6%.

## 4. Discussion

The present study evaluated the relationship between OSA severity, circulating renalase concentration, total antioxidant status, conventional cardiovascular risk factors, and the anatomical severity of CAD assessed by CCTA in patients with suspected OSA. Several principal findings emerged. First, suspected significant CAD, defined as CAD-RADS ≥ 3, was present in 22.6% of the study population. Second, patients with AHI values at or above the median, lower renalase concentrations, and lower TAS were more frequently classified as CAD-RADS ≥ 3. Third, in logistic regression analysis, older age, higher systolic blood pressure, higher total cholesterol concentration, and lower TAS were independent predictors of CAD-RADS ≥ 3. Finally, in multivariable linear regression analysis, higher pack-years of smoking, higher AHI, and lower circulating renalase concentration were independently associated with lower TAS. Taken together, these findings suggest that impaired antioxidant defense may represent an integrative biological correlate of coronary atherosclerotic burden in patients with suspected OSA, linking traditional cardiovascular risk factors, sleep-disordered breathing severity, and renalase-related redox regulation.

The relationship between OSA and CAD reported in the literature is consistent with, but not uniformly identical to, the pattern observed in the present study. OSA is widely recognized as a cardiovascular risk condition, particularly in patients with hypertension, arrhythmias, heart failure, stroke, and CAD, but its association with coronary outcomes is influenced by disease severity, hypoxemic burden, age, sex, obesity, and coexisting metabolic risk factors [10,26]. Recent data also emphasize that sleep fragmentation and arousal-related indices may provide cardiovascular information beyond AHI alone, particularly through links with sympathetic activation, blood pressure dysregulation, endothelial dysfunction, and coronary artery calcification burden [27]. Beyond conventional metabolic risk factors, pilot data indicate that severe OSA may be associated with alterations in circadian clock-related proteins involved in glucose metabolism, supporting the broader concept that OSA-related cardiovascular risk reflects a complex hypoxic, metabolic, and circadian phenotype [28]. In the Sleep Heart Health Study, OSA was associated with incident coronary heart disease in selected subgroups, particularly younger men, whereas the overall association with coronary events was less consistent than for incident heart failure [26]. Imaging studies provide more direct evidence linking OSA with coronary atherosclerosis. A systematic review of imaging-based studies reported associations between OSA and measures of coronary atherosclerosis, including coronary artery calcium score and plaque burden, while emphasizing heterogeneity in study design, imaging protocols, and confounder adjustment [29]. CCTA-based studies are particularly relevant to the present analysis. Kent et al. showed that OSA severity was associated with greater coronary plaque burden assessed by CCTA, and Sharma et al. reported an independent association between OSA and non-calcified coronary plaque [30,31]. More recent data from individuals without known cardiovascular disease also suggest that established or high-risk OSA is associated with a higher prevalence of coronary plaque on CCTA [32]. In this context, the present findings support the concept that the severity of sleep-disordered breathing, rather than the dichotomous diagnosis of OSA alone, may be clinically relevant for anatomical CAD assessment. This is reflected by the higher frequency of CAD-RADS ≥ 3 among patients with AHI values at or above the median, whereas the comparison based only on the presence versus absence of OSA showed a borderline association.

The association between lower TAS and CAD-RADS ≥ 3 observed in the present study is biologically plausible and generally consistent with the concept that impaired antioxidant defense accompanies coronary atherosclerosis. Redox imbalance may contribute to endothelial dysfunction, reduced nitric oxide bioavailability, lipid oxidation, vascular inflammation, and plaque progression, all of which are central processes in atherogenesis [12,13]. However, the clinical literature on global antioxidant capacity in CAD is not uniform. Some studies have reported altered oxidant-antioxidant profiles in patients with CAD, whereas others have shown that total antioxidant capacity is strongly influenced by conventional risk factors, metabolic status, uric acid, triglycerides, blood pressure, smoking, and other confounders [33,34]. Consistent with the broader concept of context-dependent antioxidant impairment in ischemic heart disease, lower activity of paraoxonase-1, an enzyme with antioxidant and anti-atherosclerotic properties, has been reported in patients with ischemic heart disease and concomitant periodontitis compared with those with ischemic heart disease and healthy periodontal status [35]. In a study of patients undergoing coronary angiography, calculated total antioxidant status was weakly correlated with angiographic CAD severity but was not an independent determinant of CAD after adjustment, underscoring the complexity of interpreting systemic antioxidant indices [36]. Therefore, our finding that lower TAS independently predicted CAD-RADS ≥ 3 should not be interpreted as evidence that reduced antioxidant capacity directly causes coronary stenosis. Rather, TAS may represent an integrative marker of systemic redox burden, reflecting the cumulative influence of traditional cardiovascular risk factors, smoking exposure, sleep-disordered breathing, and renalase-related antioxidant–redox pathways. This interpretation is consistent with population-based cardiovascular risk research in Poland, in which classical ASCVD risk factors have been linked conceptually with endothelial dysfunction, oxidative stress, inflammatory responses, and lipid-related pathways [37]. In line with this interpretation, lower total antioxidant capacity has been reported in smokers than in non-smokers, although available data include salivary rather than circulating antioxidant measurements and therefore should be extrapolated with caution [38]. This interpretation is also consistent with the failure of nonspecific antioxidant supplementation to produce consistent cardiovascular benefit in clinical trials, despite strong mechanistic evidence linking oxidative stress with atherosclerosis [39].

The present findings also need to be interpreted in the context of the heterogeneous literature on renalase and CAD. Renalase was initially described as a circulating kidney-derived flavoprotein involved in cardiovascular regulation, but subsequent biochemical studies suggested that its catalytic activity is more closely related to α-NAD(P)H oxidase/anomerase function, providing a mechanistic rationale for linking renalase with redox homeostasis rather than only catecholamine metabolism [21,22]. In patients undergoing coronary angiography, He et al. reported lower plasma renalase concentrations in patients with CAD than in controls and lower concentrations in patients with more extensive coronary stenosis and higher SYNTAX scores [40]. This direction is consistent with the present study, in which lower renalase concentrations were associated with lower TAS and patients with renalase concentrations below the median were more frequently classified as CAD-RADS ≥ 3. However, other clinical studies indicate that circulating renalase may behave differently depending on renal function, endothelial dysfunction, disease stage, and prior coronary intervention. In patients with established CAD, renalase interacted with chronic kidney disease in relation to endothelin-1 concentrations, suggesting a context-dependent association with endothelial activation [24]. In another cohort, higher serum renalase after percutaneous coronary intervention was associated with subsequent cardiovascular events and mortality, indicating that increased renalase may also reflect adverse neurohumoral, renal, or vascular stress in advanced disease states [41]. Therefore, in the present population of patients with suspected OSA and without chronic renal failure, lower renalase should be interpreted primarily as a marker associated with impaired antioxidant–redox balance rather than as a standalone causal determinant of CAD severity. This interpretation is further supported by data showing that lower blood renalase concentrations coexist with conventional cardiovascular risk factors, including obesity, smoking, and low physical activity [42]. However, the present observational study cannot determine whether renalase has a causal role in antioxidant defense or coronary atherosclerosis, and this mechanistic interpretation requires confirmation in experimental studies.

From a clinical perspective, the present findings should be interpreted cautiously. TAS and circulating renalase concentration should not currently be regarded as ready-to-use biomarkers for routine cardiovascular risk stratification in patients with suspected OSA. Their use remains exploratory because of the observational cross-sectional design, modest sample size, single-time-point biomarker assessment, and lack of external validation. Nevertheless, lower TAS and lower renalase concentration may characterize a redox-related phenotype associated with greater anatomical CAD burden on CCTA. Future prospective studies are required to determine whether these biomarkers provide incremental prognostic value beyond established cardiovascular risk factors, polysomnographic indices, and CCTA-derived parameters.

The present study has several strengths. First, it integrated objective sleep assessment by polysomnography with non-invasive anatomical evaluation of CAD using CCTA, rather than relying on symptom-based OSA screening or indirect cardiovascular risk estimation. Second, CAD severity was classified using CAD-RADS, which provides a standardized and clinically interpretable framework for reporting coronary stenosis severity on CCTA. Third, the study combined conventional cardiovascular risk factors with biochemical markers reflecting antioxidant–redox balance, including TAS and circulating renalase concentration. This allowed the analysis to move beyond a purely risk-factor-based model and to explore a biologically plausible pathway linking sleep-disordered breathing, antioxidant defense, and coronary atherosclerotic burden. Fourth, the use of multivariable regression models enabled simultaneous assessment of traditional risk factors, OSA severity, TAS, and renalase-related variables, thereby reducing, although not eliminating, the risk of confounding in the interpretation of the observed associations. Finally, the study was conducted in a clinically relevant population of patients with suspected OSA referred for CCTA, which increases the practical relevance of the findings for cardiovascular risk assessment in sleep-disordered breathing.

Several limitations should be acknowledged. First, the study was observational and cross-sectional; therefore, the identified associations cannot establish causality or temporal relationships between OSA severity, renalase concentration, TAS, and CAD severity. Second, the sample size was modest, and the number of patients classified as CAD-RADS ≥ 3 was limited, which may reduce statistical power, particularly in subgroup and multivariable analyses. Therefore, the present findings should be interpreted as exploratory and hypothesis-generating. Third, the study included patients with suspected OSA and clinical indications for CCTA, which increases clinical relevance but limits generalizability to the general population, asymptomatic individuals, or patients with previously established CAD. Fourth, CAD severity was assessed anatomically using CCTA and CAD-RADS, without systematic functional ischemia testing or invasive angiographic confirmation; therefore, CAD-RADS ≥ 3 should be interpreted as suspected anatomically significant CAD rather than definitive flow-limiting or functionally significant coronary disease. Fifth, OSA was characterized primarily by AHI, whereas other potentially relevant metrics, including hypoxic burden, oxygen desaturation indices, arousal burden, sleep architecture, and symptom phenotype, were not incorporated into the main models. This is relevant because polysomnographic and biochemical studies in patients with suspected OSA and hypertension indicate that sleep architecture, oxygen saturation, arousal-related indices, uric acid, magnesium, glucose, and inflammatory markers may differ according to hypertensive status and may provide additional information beyond AHI alone [43]. Sixth, TAS and renalase were measured at a single time point, and these biomarkers may be influenced by diet, medications, renal function within the non-CKD range, inflammatory status, metabolic factors, and other unmeasured confounders. Therefore, renalase and TAS should be interpreted as exploratory biomarkers rather than clinically validated tools for risk stratification. Finally, the ROC analyses should be regarded as exploratory, because the proposed cut-off values were derived from the same cohort and require external validation before clinical use. These ROC-derived thresholds should not be regarded as normative values or diagnostic criteria, particularly because established population-based reference ranges for circulating renalase are not available. They were estimated only to assess sensitivity and specificity and to determine the best discriminatory performance within this dataset.

## 5. Conclusions

Higher exposure to tobacco smoke, more severe obstructive sleep apnea, lower renalase concentration, and reduced total antioxidant status were associated with more advanced anatomically defined coronary artery disease on CCTA. These associations were observed together with established cardiovascular risk factors, including older age, higher systolic blood pressure, and higher total cholesterol concentration. The findings should be interpreted as exploratory and require confirmation in larger prospective cohorts.

## Figures and Tables

**Table 1:** Characteristics of the study group (*n* = 93).

Parameter	
Age (years) ^a^	49.82 ± 13.76
Gender ^b^ Men Women	52.7 47.3
BMI (kg/m^2^) ^a^ Overweight ^b^ Obesity ^b^	28.93 ± 5.08 37.6 44.1
Systolic blood pressure (mmHg) ^a^ Diastolic blood pressure (mmHg) ^a^ Arterial hypertension ^b^	138.39 ± 20.75 88.60 ± 11.66 36.6
Total cholesterol (mg/dL) ^a^ Triglicerydy (mg/dL) ^a^ Dyslipidemia ^b^	192.53 ± 41.99 103.08 ± 31.15 30.1
Glucose (mg/dL) ^a^ Diabetes mellitus ^b^	102.34 ± 16.39 6.4
Smoking ^b^ Pack-years of smoking ^a^	26.9 59.40 ± 35.83
AHI (/h) ^a^ Obstructive sleep apnea ^b^ Mild Moderate Severe	16.57 ± 17.17 73.1 33.3 19.4 20.4
Renalase (ng/mL) ^a^	190.33 ± 212.59
TAS (mM) ^a^	1.16 ± 0.33

^a^ arithmetic mean ± standard deviation, ^b^ percentages, AHI—apnea–hypopnea index, BMI—body mass index, TAS—total antioxidant status.

**Table 2:** Coronary computed tomography angiography parameters in the study group (*n* = 93).

**Parameter**	
CACS ^a^ CACS 0 ^b^ CACS 1–10 ^b^ CACS 11–100 ^b^ CACS 101–400 ^b^ CACS >400 ^b^	102.11 ± 200.92 43.0 14.0 17.2 17.2 8.6
CAD-RADS ^b^ 0 1 2 3 4 5 ≥3	41.9 21.5 14.0 16.1 4.3 2.1 22.6
LV EDV (mL) ^a^ LV ESV (mL) ^a^ LV SV (mL) ^a^ LV EF (%) ^a^ LVM (g) ^a^	147.50 ± 47.74 46.16 ± 19.21 101.62 ± 32.78 69.16 ± 7.03 114.89 ± 30.01

^a^ arithmetic mean ± standard deviation, ^b^ percentages, CACS—coronary artery calcium score, CAD—coronary artery diseases, EDV—end-diastolic volume, EF—ejection fraction, ESV—end-systolic volume, LVM—left ventricular mass, LV—left ventricle, RADS—reporting and data system, SV—stroke volume.

**Table 3:** Severity of coronary artery disease assessed by multidetector computed tomography angiography in the study subgroups.

Differentiation Criterion	Subgroup	CAD-RADS ≥ 3 ^b^	*p*
Obstructive sleep apnea ^b^	A: OSA (*n* = 68)	27.9	0.06
	B: without OSA (*n* = 25)	8.0	
Obstructive sleep apnea severity ^b^	A1: severe OSA (*n* = 31)	36.8	0.34
	A2: moderate OSA (*n* = 18)	33.3	
	A3: mild OSA (*n* = 19)	19.3	
Median of the AHI variable (=10.10/h) ^a^	C: AHI ≥ median (*n* = 47)	31.9	0.03
	D: AHI < median (*n* = 43)	13.0	
Median of the renalase variable (=64.03 ng/mL) ^a^	E: renalase ≥ median (*n* = 47)	12.8	0.02
	F: renalase < median (*n* = 46)	32.6	
Median of the TAS variable (=1.14 mM) ^a^	G: TAS ≥ median (*n* = 49)	10.2	0.02
	H: TAS < median (*n* = 44)	36.4	

^a^ arithmetic mean ± standard deviation, ^b^ percentages, AHI—apnea–hypopnea index, CAD—coronary artery diseases, OSA—obstructive sleep apnea, RADS—reporting and data system, TAS—total antioxidant status.

**Table 4:** Results of regression analysis in the study group (*n* = 93).

A. Estimation of the Logistic Regression Analysis Model for the Dependent Variable CAD-RADS ≥ 3.	Model for: Probability of CAD-RADS ≥ 3				
	Intercept	Age (Years)	Systolic Blood Pressure (mmHg)	Total Cholesterol (mg/dL)	TAS (mM)
Regression coefficient	−17.015	0.093	0.059	0.035	−4.642
SEM of Rc	6.013	0.039	0.027	0.013	1.957
*p* value	0.006	0.020	0.029	0.009	0.020
Odds ratio (for unit change)	0.001	1.097	1.061	1.035	0.010
Confidence interval −95%	0.001	1.015	1.006	1.009	0.001
+95%	0.006	1.186	1.118	1.063	0.471
B. Estimation of the backward stepwise multivariable regression analysis model for the dependent variable TAS (mM).	Model for: TAS (mM)				
	Intercept	Pack-years of smoking	AHI (/h)	Renalase (ng/mL)	
Regression coefficient	1.328	−0.003	−0.005	0.001	
SEM of Rc	0.116	0.001	0.003	0.001	
*p* value	0.001	0.010	0.021	0.036	

AHI—apnea–hypopnea index, CAD—coronary artery diseases, RADS—reporting and data system, SEM of Rc—standard error of the men of regression coefficient, TAS—total antioxidant status.

**Table 5:** The sensitivity and specificity of selected discriminators of CAD-RADS ≥ 3 in the study group (*n* = 93).

Target Variable	CAD-RADS ≥ 3	CAD-RADS ≥ 3	CAD-RADS ≥ 3	CAD-RADS ≥ 3
Predictor variable	AHI > 5/h	AHI > 14.2/h	Renalase < 60.10 ng/mL	TAS < 1.18 mM
Sensitivity	0.319	0.667 *	0.597	0.500
Specificity	0.905 **	0.619	0.667	0.857
Accuracy	0.452	0.656 ***	0.613	0.581
Positive predictive values	0.920	0.857	0.860	0.923
Negative predictive values	0.279	0.351	0.326	0.333
Likelihood ratios positive	3.354	1.750	1.792	3.500
Likelihood ratios negative	0.752	0.538	0.604	0.583

* highest sensitivity, ** highest specificity, *** highest accuracy, AHI—apnea–hypopnea index, CAD—coronary artery diseases, RADS—reporting and data system, TAS—total antioxidant status.

## Data Availability

The data are available from the authors upon reasonable request.

## Abbreviations

The following abbreviations are used in this manuscript:

AHI	apnea–hypopnea index
CACS	coronary artery calcium score
CAD	coronary artery disease
CAD-RADS	coronary artery diseases—reporting and data system
CCT	cardiac computed tomography
CCTA	coronary computed tomography angiography
CT	computed tomography
EACVI	European Association of Cardiovascular Imaging
EBCR	European Board of Cardiovascular Radiology
ELISA	enzyme-linked immunosorbent assay
OSA	obstructive sleep apnea
PSG	polysomnograpy
TAS	total antioxidant status
